# Adaptive visual detection of industrial product defects

**DOI:** 10.7717/peerj-cs.1264

**Published:** 2023-03-15

**Authors:** Haigang Zhang, Dong Wang, Zhibin Chen, Ronghui Pan

**Affiliations:** 1 Shenzhen Polytechnic, Shenzhen, China; 2 University of Science and Technology Liaoning, Anshan, China

**Keywords:** Model-agnostic meta-learning, Industrial visual inspection, Convolutional neural network

## Abstract

Visual inspection of the appearance defects on industrial products has always been a research hotspot pursued by industry and academia. Due to the lack of samples in the industrial defect dataset and the serious class imbalance, deep learning technology cannot be directly applied to industrial defect visual inspection to meet the real application needs. Transfer learning is a good choice to deal with insufficient samples. However, cross-dataset bias is unavoidable during simple knowledge transfer. We noticed that the appearance defects of industrial products are similar, and most defects can be classified as stains or texture jumps, which provides a research basis for building a universal and adaptive industrial defect detection model. In this article, based on the idea of model-agnostic meta-learning (MAML), we propose an adaptive industrial defect detection model through learning from multiple known industrial defect datasets and then transfer it to the novel anomaly detection tasks. In addition, the Siamese network is used to extract differential features to minimize the influence of defect types on model generalization, and can also highlight defect features and improve model detection performance. At the same time, we add a coordinate attention mechanism to the model, which realizes the feature enhancement of the region of interest in terms of two coordinate dimensions. In the simulation experiments, we construct and publish a visual defect dataset of injection molded bottle cups, termed BC defects, which can complement existing industrial defect visual data benchmarks. Simulation results based on BC defects dataset and other public datasets have demonstrated the effectiveness of the proposed general visual detection model for industrial defects. The dataset and code are available at https://github.com/zhg-SZPT/MeDetection.

## Introduction

Industrial surface defect detection is intuitively important for industrial production, which can not only help enterprises to improve product quality to meet the growing needs of consumers, but also help enterprises to locate problems in a timely manner to reduce production costs (Xiao et al., 2021). Only relying on human power to complete visual inspection cannot guarantee efficient industrial production requirements. Human subjective consciousness and long-term eye fatigue are prone to product misdetection and missed detection (Bhatt et al., 2021). Using computer vision technology to solve the intelligent detection of appearance defects of industrial products has always been the goal pursued by industry and academia.

Relying on traditional image processing technology to solve the problem of visual inspection of industrial defects has a long research history, which can be divided into two types of research methods (Carrasco et al., 2021). On the one hand, the specific feature extractors are manually designed to extract pixel-wise and structure-wise image features, which then are fed into the traditional classifier (KNN, SVM, BP *etc*.) for defect identification (Xue-Wu et al., 2011). However, if the extracted features are not precise enough, the judgments made by relying on them are bound to be inaccurate. Meanwhile, if the extracted features are not fine enough and the dimensionality of the feature space is too large, the complexity of the subsequent discriminative algorithm may be very high. On the other hand, the feature difference between the sample to be tested and the normal template is calculated by means of template matching, so as to determine whether there are defects on the sample to be tested. However, the choice of template and the limitations of the matching algorithm often affect model performance. In general, traditional industrial image processing methods rely on manual design features, and the generalization ability is poor. The relatively fixed feature extractors make the detection model more limited in application.

In the context of big data, deep learning techniques represented by a convolutional neural network (CNN) has achieved extensive development and progress in the field of computer vision and pattern recognition. Using the learned features to replace the handcrafted features, a CNN algorithm can map the pixel space features to high-layer semantic representation based on a series of operations consisting of convolution and pooling. There are already many deep neural network models of deep learning, like AlexNet, VggNet, GoogLeNet, ResNet, *etc*. (Ma et al., 2021), which lays a solid foundation for industrial visual inspection research. The application of CNN in industrial visual inspection can refer to some review works (Chen et al., 2021, Qi, Yang & Zhong, 2020).

The tasks of visual detection of defects in industrial products have some unique characteristics that lead us to fully consider these factors when designing CNN based detection models:

- Industrial defect datasets present a serious sample non-equilibrium phenomenon. In the production process of industrial products, the occurrence of defects is a small probability event. Therefore, the number of sample classes is unbalanced. This phenomenon can easily lead to model overfitting.
- Industrial defect detection belongs to the multi-objective and multi-scale detection tasks. There are various types of defects and multi-scale differences between the same types. From the computer vision perspective, such multi-objective and multi-scale detection tasks require higher demand on the performance of the detection model.
- The occurrence of industrial defects has strong randomness. The type, location, size, and severity of industrial defects cannot be predetermined, presenting great randomness, and the collected samples are difficult to meet the marginal effect of the defect feature data, which seriously affects the performance of the detection model.

Considering the unbalanced phenomenon of industrial defect data distribution, it is unrealistic and insufficient to solve the above problems by complicating the model or deepening the number of network layers. There are many ways (Lin et al., 2017; Elkan, 2001; Fernández et al., 2018) to address data imbalance problems. Transfer learning is a good choice to solve the problem of insufficient data or imbalanced data distribution. By training the model on other large datasets and fine-tunning on the target detection task set, the model performance can often be improved without too much data. Some works (Abu et al., 2021; Liu et al., 2021) implement knowledge transfer for industrial defect detection based on public datasets, such as ImageNet (Krizhevsky, Sutskever & Hinton, 2017), COCO (Lin et al., 2014), *etc*. Additionally, there are some works arguing that it is more reasonable to implement knowledge transfer based on similar industrial defect datasets (Zhao et al., 2020; Ri-Xian, Ming-Hai & Xian-Bao, 2015; Wang & Xiao, 2021). There are huge differences in the characteristics of different industrial defects. For example, black spots on the surface of white products and white spots on the surface of black products belong to two different detection tasks. This phenomenon is called as cross-dataset bias. Transfer learning must effectively solve the cross-dataset bias problem in order to ensure the effectiveness of target detection knowledge reuse. Even if the detection knowledge transfer is realized between different industrial defect visual inspection tasks, due to the different production process of industrial products, defect types and other factors, simple knowledge transfer often not only fails to achieve effective initialization weight settings for the detection model, but also misguides the optimization direction.

**Figure 1:**
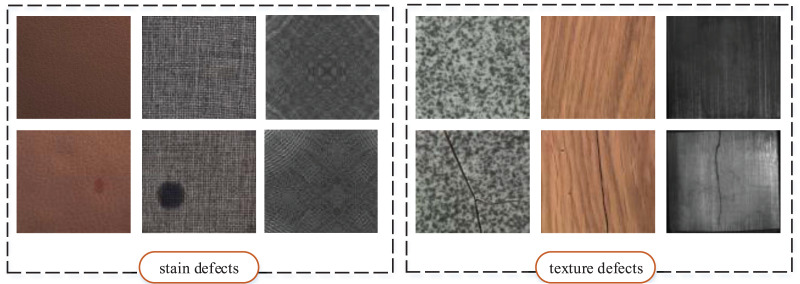
Two types of surface defects of industrial products. Images taken from Bergmann et al. (2020a) (License: CC-BY-NC-SA 4.0).

Generally, the types of appearance defects of industrial products can be roughly divided into two categories, as shown in Fig. 1. Stain defects mainly refer to abnormal color jumps on the surface of products, while texture defects generally aim at products with textured features on the surface, which refer to the phenomena that have a destructive effect on the texture performance of products. The similarity of industrial defects motivates us to think that, whether is it possible to establish a unified network framework for industrial defect feature extraction with strong application generalization based on known industrial defect datasets. Such a feature extraction framework can learn the common features of industrial defects, at least effectively for stain and texture defects, which is the main motivation of our work.

Transfer learning only considers the best results to be achieved on the current training data, resulting to lead to negative knowledge transfer possibly. Meta-learning (Finn, Abbeel & Levine, 2017) does not directly teach the model how to solve a given task but learning to learn. For multiple tasks, the model under the meta-learning training strategy does not pursue performance under a specific task, but is committed to extracting the common characteristics of data distribution in different task sets, and its performance is balanced in multiple task sets. Model-agnostic meta-learning (MAML) (Finn, Abbeel & Levine, 2017) is a typical representative of meta-learning. For MAML training strategy, in a training cycle, multiple task sets are fed into the model, which is updated with the error sum of the model acting on these tasks. We believe that a general-purpose industrial defect feature extraction model can be constructed by using the MAML-based training strategy on the public industrial defect dataset, which is the main contribution of this article. To the best of our knowledge, it is the first time to apply the MAML training strategy to the construction of the visual inspection model for industrial defects.

MAML seeks common target features across multiple training datasets, which requires similarities across tasks. Although we can attribute most industrial defects to stains or texture defects, there are large differences between defects of the same type. In terms of stain defects, white stains on black surfaces and black stains on white surfaces belong to two distinct detection tasks. We hope that the model can pay attention to the feature difference changes on the surface of industrial products, instead of determining whether the products are good or not based on color change or other specific features. In the proposed model, in order to further unify and highlight defect features, Siamese network (Wu et al., 2019; Luan, Jing & Zuo, 2021) is used to reinforce feature differences. The Siamese network is configured with dual-stream channels, in which the main channel is used for feature extraction of defect-free samples, and the samples to be tested are fed into the secondary channel as input. After the feature difference calculation, the Siamese network can ensure that there is no response when normal samples are input to the secondary channel, and when defective samples are set as input, the activation response is realized.

In this article, a general and adaptive visual recognition model for appearance defects of industrial products, termed MeDetection (Mete Detection), is proposed based on MAML training strategy. MeDection model takes the Siamese network as input, followed by the 4Conv (Finn, Abbeel & Levine, 2017) backbone to achieve defect feature extraction. The 4Conv framework is commonly used in industrial visual inspection models as the backbone for feature extraction (Sun et al., 2019). In order to improve the accuracy of the MeDetection model for unified feature extraction of industrial defects, the coordinate attention (CA) is embedded into the network to achieve feature enhancement at the location of the defect. It is worth mentioning that MeDetection method mainly deals with industrial defect recognition, and it can also obtain the location of defects with weakly supervised localization algorithm (like Grad-CAM (Selvaraju et al., 2017) in this article). In addition, in this article, a new visual dataset of industrial product defects is released. We constructed a defect dataset of industrial injection-molded bottle caps by means of collection, named BC defects dataset, which contains eight types of defects. The BC defects dataset contains 3,008 images and considers the case of multiple defects in the same sample. BC defects dataset is a good complement to the current industrial defect visual benchmarks. In summary, the contributions of this article are as follows:

1. Based on the MAML training strategy, a general and adaptive visual detection model, named MeDetection, for industrial defects is constructed, which can achieve unified feature extraction for industrial defects and facilitate knowledge transfer in new industrial inspection tasks;
2. The Siamese network realizes the common feature extraction of the two-stream branch, and the feature difference highlights the response of the defect in the feature space;
3. Coordinate attention is embedded into the MeDetection model, which further improves the performance of industrial defect detection;
4. We publish a visual dataset of injection-molded bottle cap defects, named BC defects. Moreover, the validity of the MeDetection model is verified by using the BC defects dataset and other publicly available industrial defect datasets.

The structure of this article is as follows. Section 1 presents the related work. Section 2 introduces the defect detection model proposed in this article. Section 3 introduces the BC defects dataset. Section 4 shows the simulation results and Section 5 is the conclusion.

## Related work

### Traditional visual detection methods

Traditional visual detection methods mainly relies on manually designed features to complete defect recognition, which can be divided into two categories: the texture feature-based methods and the color feature-based methods.

- *The texture feature-based methods*: The texture feature-based methods are mainly based on the grayscale distribution of pixels and their spatial neighborhoods for anomaly detection, which can be further classified into three categories: statistical methods, signal processing methods, and model methods. For statistical methods (Song et al., 2015), the main idea is to describe various statistical characteristics of the distribution of gray values. Then detection is performed by artificially setting thresholds. The signal processing methods (Tsai, Wu & Li, 2012) regard the image as a two-dimensional signal and analyze the image from the perspective of signal filter design. The traditional modeling method is to build detection models for specific tasks, and the common detection methods are mainly MRF (Markov random field) models (Luthon, Popescu & Caplier, 1994) and fractal models (Xu, 2015) *etc*.
- *The color feature-based methods*: Stain defects on the surface of industrial products can cause color jumps. The color feature-based methods mainly uses color histograms (Prasitmeeboon & Yau, 2019) to describe the proportion of different colors in an image or color moments (Li, Quan & Wang, 2020) to describe the distribution of colors to accomplish anomaly detection.

Traditional defect detection methods are mainly designed for specific tasks, and the model has poor generalization and flexibility. The feature extraction strategy based on manual setting is unstable and cannot effectively deal with the complex industrial production environment.

### Deep learning detection methods

Deep learning applies the end-to-end training strategy to enable the model to learn the defect features automatically, which can enhance the model’s generalization performance. Deep learning based methods for industrial defect detection can be divided into two main categories: supervised learning and semi-supervised or unsupervised learning.

- *Supervised learning*: Supervised industrial defect detection methods are mainly applied to situations where the defect patterns are known. The most direct and simple way is to apply CNN-based classification (Zhang, Gu & Zhang, 2021), detection (Li et al., 2021), and segmentation (Long, Shelhamer & Darrell, 2015) models to industrial defect detection tasks. The classification of the appearance quality of industrial products is the simplest and most straightforward visual inspection task. However, for some special applications, it is necessary to obtain the position information of industrial product defects, even the contour information, to improve the production process. Representative works can refer to Villa et al. (2018) and Jing et al. (2020).
- *Semi-supervised or unsupervised*: In practical industrial production, it is not easy to obtain enough defect datasets. Even the occurrence of zero-sample unknown defects is possible. The semi-supervised and unsupervised industrial defect detection methods mainly applies to the situations with unknown defects. The unsupervised industrial defect detection methods mainly rely on generative models, which believe that generators built with known samples cannot produce satisfactory results for the discriminator when encountering unknown defects. The most commonly applied reconstruction methods are autoencoder (AE) (Alahmadi, Alkhraan & BinSaeedan, 2022) and generative adversarial network (GAN) (Arora & Soni, 2021). The semi-supervised industrial defect detection methods, from a statistical point of view, consider that the distribution of abnormal samples in the feature space is inconsistent with that of normal samples. In high-dimensional space, or abstract feature space, unknown sample detection can be achieved by setting the data distribution discriminator. Typical methods can be referred in SPADE (Cohen & Hoshen, 2020), PADIM (Defard et al., 2021).

Industrial defect detection models pursue application generalization and rapidity. The strong adaptive feature extraction network for industrial defects is the key. To the best of our knowledge, there is currently no research focusing on general-purpose feature extraction models for industrial defects.

### CNN and meta learning

With the increase in the amount of data and the improvement of computing power, deep neural networks (DNNs) have shown strong performance in the field of machine learning. CNN is the most typical representative of DNNs, which can allow image-level input and realize local feature extraction of images. Feature extraction based on convolution operation is the most important component in CNN. In a specific layer of convolution operation, the weight-shared convolution kernel traverses the entire image, and serves as a feature extractor to highlight local target features. The training process of CNN model is the process of determining parameters of convolution function, which is composed of the trainable parameters $w = \left( {{w_1},{w_2}, \cdot \cdot \cdot ,{w_L}} \right)$ with random initialization. The trainable or learning parameters can be discriminatively determined by sample data $\left( {{x_i},{y_i}} \right)$, for $i = 1,2, \cdot \cdot \cdot ,n$, so that $\sum\nolimits_n {\ell \left( {f\left( {{x_i},w} \right),{y_i}} \right)} \to \min$, where $\ell \left( \cdot \right)$ is the loss function. Convolutional layer is an important part of CNN. The convolutional layer usually consists of an input feature map, an output feature map, and a learnable convolutional kernel. The input feature map is a three-dimensional structure with a shape of $M \times N \times D$, and consists of *D* feature maps of size $M \times N$. The shape of the output feature map is similar to that of the input feature map, except that the size of each dimension may vary. Generally, one can using the back-propagation way to calculate the optimization gradients of learning parameters, and using a specific optimizer (*e.g*., Stochastic Gradient Descent or Adam) can lead CNN network gradually converge and obtain the satisfied learning parameters.

Meta learning is a type of transfer learning that aims to learn a general feature extractor from multiple datasets. Finn, Abbeel & Levine (2017) firstly proposed the ordinary MAML algorithm in 2017, which has undergone a great deal of follow-up research. Antoniou, Edwards & Storkey (2018) proposed MAML++, which improved MAML using an annealing algorithm to improve the generalization performance, convergence speed and computational power of MAML. Baik et al. (2021) made improvements to the loss function of MAML and proposed the first learnable loss function MeTAL, which enhanced the generalization ability of MAML. Zhou et al. (2021) theoretically derived an upper bound on the error of MAML and pointed out that the improvement in generalization performance for the target task is greater when the training task and the target task are more similar. Raghu et al. (2019) experimentally found that the generalization ability of MAML mainly comes from feature reuse, and proposed ANIL to substantially reduce the computational effort while ensuring generalization.

The models with CNN structure generally belong to the task-driven training methods, which seek to achieve satisfactory fitting accuracy under specific tasks. In contrast, the input of MAML is multiple task sets, and the training of MAML based models requires the balance of performance on multiple target tasks. MAML is the main training strategy of the proposed MeDetection model in this article, and the trained feature extraction network has a strong ability to extract general features for industrial defects.

## Medetection model

**Figure 2:**
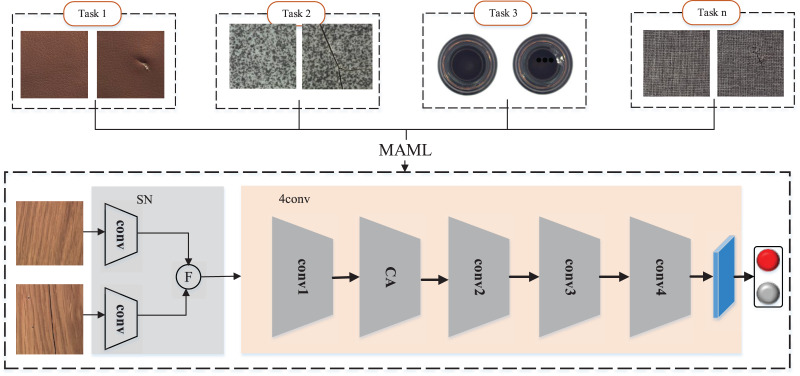
MeDetection model. SN stands for Siamese network, which is applied to calculate the difference features. *F* stands for the feature difference extraction module. The 4conv network is set as the main feature extraction backbone, while MAML based training strategy is applied to optimize the detection model. The CA module embedded in the feature extraction backbone further strengthens the defect features from the perspective of coordinate attention. Images taken from Bergmann et al. (2020a) (License: CC-BY-NC-SA 4.0).

The MeDetection model as shown in Fig. 2 focuses on building a general and adaptive visual detection framework for industrial defects. In order to realize the effective unification of industrial defect feature representation, we construct a feature difference extraction module based on Siamese network (SN). The MAML-based training strategy is the key to the optimization of the MeDetection model, which takes different industrial defect visual detection tasks as input, and tries to build a unified and generalized feature extraction framework for different defect types. In order to better highlight the defect features, the CA module is embedded in the recognition model, which can achieve different attention responses to visual features from the row and column coordinate dimensions.

### Feature difference extraction module

It can be seen from Fig. 1 that the appearance defects of industrial products can be regarded as feature differences on the normal appearance, which gives us a conclusion to judge whether there are appearance defects on industrial products by detecting feature differences (Zhang et al., 2023). In the MeDetection model proposed in this article, the Siamese network serves the purpose of extracting difference features. Different from the traditional CNN structure, the Siamese network consists of parallel two-stream branches. As shown in Fig. 2, two branches input normal samples and test samples respectively, where the test samples contain normal and defective data. The feature extraction weights of the two branches are shared. The output of the Siamese network is obtained by the difference features of the two branches, such that

(1) $${x_o} = abs\left( {Con{v_\theta }\left( {{x_1}} \right) - Con{v_\theta }\left( {{x_2}} \right)} \right)$$

where ${x_1},{x_2}$ represent the dual stream inputs, ${x_o}$ is the feature difference output. $Con{v_\theta }\left( \cdot \right)$ represents the feature extractor with $\theta$ as learning parameters, and $abs\left( \cdot \right)$ obtains the absolute values to ensure the non-negativity of the features.

### Model training and optimization

Unlike task-driven pattern recognition models, the proposed MeDetection model is trained on multiple industrial defect visual inspection tasks. The types of industrial defects can be roughly divided into stains and texture defects, and the feature difference extraction module further improves the consistency of defect expression. We hope that the MeDetection model has generalized feature extraction capabilities and can adapt to different types of industrial defect recognition tasks.

The MAML-based training strategy is applied in MeDetection model. MAML seeks to achieve a balance of model performance on multiple task sets, that is, MAML does not seek to achieve the optimal model on a specific task, but hopes that the model can achieve comprehensive optimality on multiple task sets. The optimization function of MAML is as follows:

(2) $$L\left( {{f_\theta }} \right) = \sum\limits_{i = 1}^{N}{\ell _{{T_i}}}{( \,f_{\theta}{_{i}^{\prime}})}$$

where $L\left( {{f_\theta }} \right)$ represents the loss function of the model ${f_\theta }$ with $\theta$ as the learning parameter, which is equal to the sum of the loss functions ${({\ell _{{T_i}}}{( f_{\theta}{_{i}^{\prime}})})}$ of the model applied to all task sets ${T_i},i = 1,2, \cdot \cdot \cdot ,N$. ${{\theta}_{i}^{\prime}}$ represents the task-specific weights generated by transferring the learning parameter $\theta$ to the $i$th task after fine-tuning operation. *N* is the number of tasks.

MAML consists of two loops: Inner loop and Outer loop. The optimal parameters for each task are calculated iteratively in the inner loop, while the outer loop updates the learning parameters for the entire model by computing the gradients relative to the optimal parameters in each new task. The pseudocode is shown in Algorithm 1. We select a variety of industrial products datasets to form the task sets $p(T)$. Specifically, $p(T)$ represents the probability distributions of all the training tasks (Finn, Abbeel & Levine, 2017). The learning rates of the inner loop and outer loop of MAML are set to $\alpha$ and $\beta$. During model optimization, the inner loop is executed firstly, and followed by the outer loop.

**Algorithm 1:** MAML

**Require:** Distribution over tasks $p\left( T \right)$
**Require:** Step size of the hyperparameters ${\alpha},\,{\beta}$
1: Randomly initialize *θ*
2: **while** not done **do**
3: Sample batch of tasks ${T_i}\sim p\left( T \right)$
4: **for** **all ** ${T_i}$ **do**
**5:** **Sample *K* datapoints** ${D_i} = \left\{ {{x^{(j)}},{y^{(j)}}} \right\}$
6: **Evaluate** ${\nabla _\theta }{\ell _{{T_i}}}\left( {{f_\theta }} \right)$ **using** *D*_*i*_ **and** ${\ell _{{T_i}}}$ **in Eq. (3)**
7: **Compute adapted parameters using Eq. (4)**
8: **Sample datapoints** $D_{i}^{\prime} = \left\{ {{x^{(j)}},{y^{(j)}}} \right\}$ **from** ${T_i}$
9: **Compute** ${\ell _{{T_i}}}(f_{\theta}{{_{i}^{\prime}}})$ **in Eq. (3) using** ${ D_{i}^{\prime}}$
10: **end for**
11: **Update *θ* using Eq. (5)**
12: **end while**

- *Inner loop*: We select *K* samples ${D_i} = \left\{ {{x^{(j)}},{y^{(j)}}} \right\}$ from the task ${T_i}$. For classification task, ${\ell _{{T_i}}}$ is computed as follows:

(3) $${\ell _{{T_i}}}({f_\theta }) = \sum\limits_{{x^{(j)}},{y^{(j)}}\sim{T_i}}^{} {{y^{(j)}}\log } \;f_{\theta} ({x^{(j)}})\\ + (1 - {y^{(j)}})\log (1 - f_{\theta} ({x^{(j)}}))$$

Then the updated gradients of model for the task ${T_i}$ can be computed as ${\nabla _\theta }{\ell _{{T_i}}}\left( {{f_\theta }} \right)$. The learning parameters $\theta _i^\prime$ for the task ${T_i}$ based on model ${f_\theta }$ can be obtained as:

(4) $$\theta _i^\prime = \theta - \alpha {\nabla _\theta }{\ell _{{T_i}}}({\,f_\theta })$$

where ${\ell _{{T_i}}}(f_{\theta} {_i^{\prime}})$ is calculated using the remaining samples $D_{i}^{\prime}$ of the task ${T_i}$ based on Eq. (3).

- *Outer loop*: During the outer loop, the model parameters $\theta$ are updated using the following equation:

(5) $$\theta \leftarrow \theta {\rm{ - }}\beta {\nabla _\theta }\sum\limits_{{T_i}\sim p\left( T \right)}{\ell _{{T_i}}}{(\,f_{\theta}{_{i}^{\prime}})}$$

### Coordinate attention

**Figure 3:**
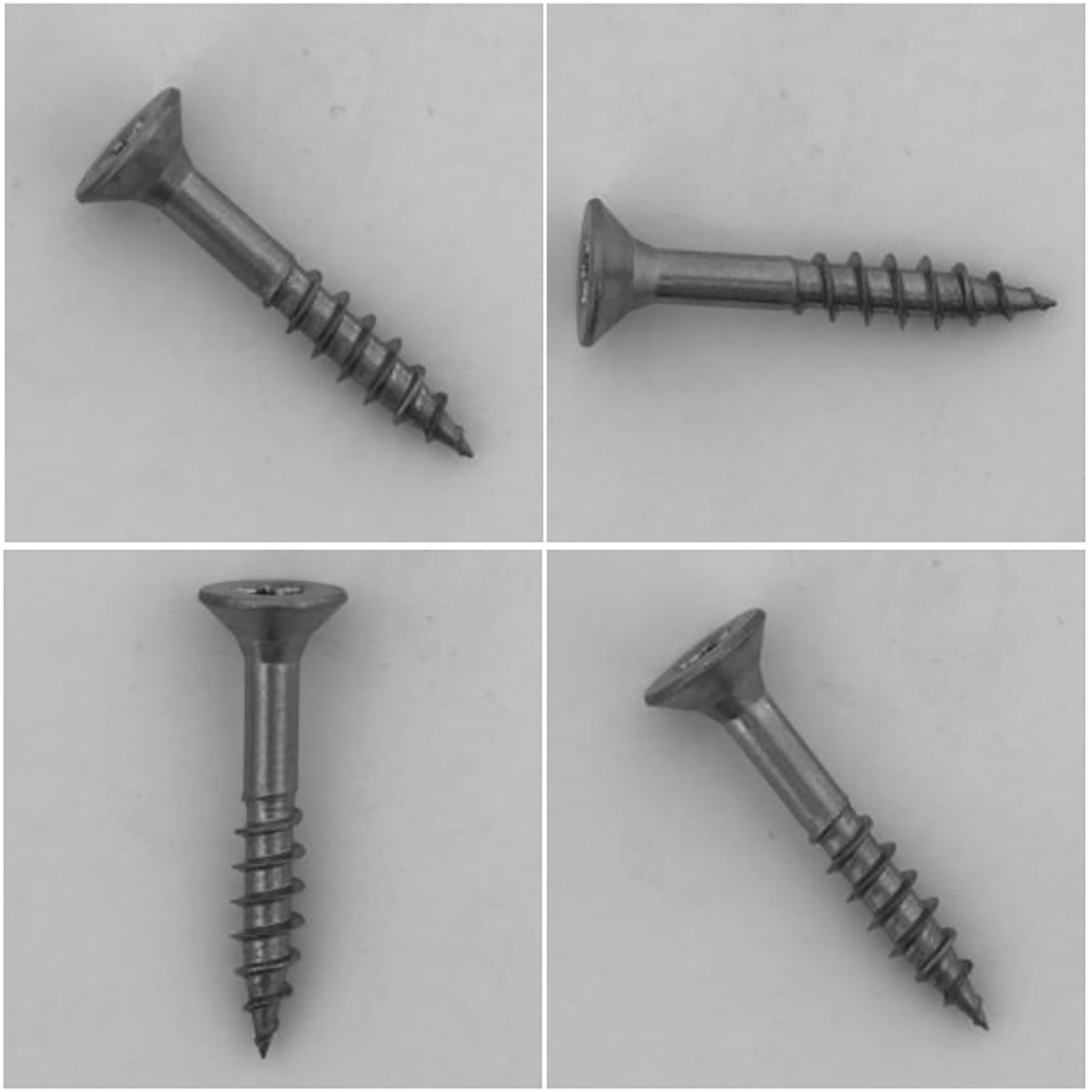
Visualization of “screw” dataset, where the samples are placed randomly. Images taken from Bergmann et al. (2020a) (License: CC-BY-NC-SA 4.0).

The defect features extracted from the SN module may contain redundant information in the fusion process, which is not conducive to the recognition of defects by the model. CA (Hou, Zhou & Feng, 2021) constructs feature mapping from the horizontal and vertical dimensions, and interacts the feature information among channels. Through automatically re-weighting the features, CA module can highlight the defect features and eliminate the redundant information. As shown in Fig. 3, for the input $X = [{x_1},{x_2},{x_3},...,{x_c}] \in {{\mathbb R}^{C \times H \times W}}$, CA uses two pooling kernels with dimensions of $(H,1)$ and $(1,W)$ to encode each channel along the horizontal coordinate and the vertical coordinate respectively. The output of the $c$-th channel at height $h$ and width $w$ can be expressed as

(6) $$\left\{ {\matrix{ {z_c^h\left( h \right) = {1 \over W}\sum\limits_{0 \le i{{\lt}}W} {{x_c}} \left( {h,i} \right)} \cr {z_c^w\left( w \right) = {1 \over H}\sum\limits_{0 \le j{{\lt}}H} {{x_c}} \left( {j,w} \right)} \cr } } \right.$$

The above two transformations enables the model to capture long-distance relationships in one direction while retaining spatial information in the other direction, which can help the network to identify defects more accurately. Then, CA module maps ${z^h}$ and ${z^w}$ to the corresponding coordinate attention weights, and reweighting operations are carried out.

The attention responses of CA module contain inter-channel information, horizontal spatial information and vertical spatial information, which can help the network to obtain the location information of defects more accurately and enhance the ability of feature extraction.

## Experimental results

### Dataset

The MeDetection model is able to learn common representations of defect features from multiple industrial defect tasks, and then implement knowledge transfer on new tasks. Therefore, we need to first prepare a cluster of industrial defect visual inspection task sets. In the experiments, DAGM (Wang et al., 2018) and MVTec (Bergmann et al., 2020a; Wieler & Tobias Hahn, 2007) datasets are applied in the training of MeDetection model. The MVTec dataset contains a total of 15 categories, five categories of which are texture-based data, and contain regular patterns (blankets, grids) and random patterns (leather, tiles, wood). The remaining 10 categories are object-based data, which contain objects with a specific appearance (bottles, metal nuts), deformable objects (cables) or objects including natural variation (hazelnut). Some of the acquired objects are in approximately aligned position (toothbrushes, capsules, and pill), the others were randomly placed (metal nuts, screws, and hazelnuts). The DAGM dataset contains 10 different types of fabric texture defects. Each class consists of 1,000 defect-free images and 150 defective images saved in grayscale eight-bit PNG format. The detailed information about the applied detect datasets is shown in Table 1.

**Table 1:** Statistics of the selected defect datasets.

Tasks	No. of normal samples	No. of anomaly samples
Sidewalk	750	150
Carpet	308	89
Walls	750	150
Zipper	272	119
Transistors	273	40
Wood	266	60
Bridges	273	40
Hazelnut	431	70
Toothbrush	72	30
Bottles	229	63
Tiles	263	84
Cables	282	92
Leather	277	92
Capsule	242	109
Pill	293	141
Screws	361	119
Grid	285	57
Metal nuts	242	93
10 types of fabric	750	150
BC defects	710	268

**Note:**

The 10 types of fabrics are the fabric data of the DAGM dataset. Due to the similarity of the types, each type of fabrics is not listed separately.

In addition, in the previous work (Zhang et al., 2023), we published a novel visual dataset on appearance defects of industrial injection molding products, BC defects. There are 3,008 samples in BC defects dataset, including 1,608 normal samples and 1,400 ones with eight kinds of appearance defects.

### Simulation details

The experiments run on a computer with NVIDIA DGX A100 SXM4 40G GPU. The experimental framework is pytorch1.9.0 with cuda 11.2. The image size is set to $256 \times 256$. Adam with parameters ${\beta _1} = 0.5$ and ${\beta _2} = 0.999$ is used as the optimizer during training. We set the batchsize as 64. The max epoch is set as 1,000, and we use the early-stopping strategy to decide to stop the model training. The learning rates of inner and outer loops are set as $\alpha ={10^{{\rm{ - }}5}}$ and $\beta ={10^{{\rm{ - }}3}}$ respectively. Since the number of each industrial product is not unique, we first divide each task dataset into a support set and a query set according to 7:3, and then randomly select 64 images from the support set in the inner loop of MAML to train the model and then randomly select 20 images from the query set to validate the model. After MAML training, MeDetection will be fine-tunned on the target task with the initial learning rate as 0.0001. The advantage of the MeDetection model proposed in this article is that it can be trained on multiple industrial defect data sets and then transfer knowledge to unknown industrial defect recognition task. During training, we remove one target task from the data sets and train the model with the remaining industrial defect data sets. During the verification process, the trained MeDetection model is fine-tuned for the target task based on the pretrained learning parameters. In order to evaluate the model performance, we follow the metrics in Akcay, Atapour-Abarghouei & Breckon (2018) and compute the area under receiver operator characteristics (AUROC), which measures the area under the true positive rate as a function of the false positive rate. The AUROC metric is not sensitive to any threshold or the percentage of anomalies in the test set. In addition, other metrics, like Recall, Precision, F1 Score and the convergence speed of model, are taken into consideration for evaluating the model in all aspects.

### Comparison experiments

In order to show the performance of MeDetection model more clearly, some excellent industrial defect detection algorithms are added to the comparative experiment. GANomaly (Akcay, Atapour-Abarghouei & Breckon, 2018) belongs to the semi-supervised defect detection *via* adversarial learning, which point out the unknown defect data lead to a larger distance metric from the learned (known) data distribution at inference time. One-NN (Nazare, de Mello & Ponti, 2018) represents the works that apply transfer learning technology into industrial defect detection, and it analyzes the usage of different feature normalization techniques on the pre-trained CNN models. DOCC (Ruff et al., 2021) is short for deep one-class classification method, which aims to deal with the unbalanced data distribution in industrial defect detection tasks. PatchSVDD (Yi & Yoon, 2020) is a long-standing algorithm used for an anomaly detection, which can search for a data-enclosing hypersphere in the kernel space, and compare the difference in data distribution between normal and defective samples. U-Student (Bergmann et al., 2020b) belongs to a powerful student–teacher framework for the challenging problem of unsupervised anomaly detection and pixel-precise anomaly segmentation in high-resolution images, where Student networks are trained to regress the output of a descriptive teacher network that was pretrained on a large dataset of patches from natural images. Anomalies are detected when the outputs of the student networks differ from that of the teacher network.

Table 2 shows the quantitative comparison results of AUROC metrics between the proposed MeDetection model with other state-of-the-arts on industrial defect detection tasks in MVTec (Bergmann et al., 2020b) datasets. The “Tasks” column represents the target task set, which needs to be eliminated during model training. MeDetection model gets the highest AUROC on six tasks (“carpet”, “leather”, “wood”, “capsule”, “pill” and “zipper”), the second best AUROC on three tasks (“grid”, “bottle” and “hazelnut”). Especially for the recognition task in “leather” dataset, MeDetection model gets 100% accuracy. The last column of Table 2 shows the average AUROC value. The MeDetection model achieves the best accuracy (94.0%), 1.5 percentage points higher than the U-Students model (Bergmann et al., 2020b), which achieves the second-best results in the overall comparison. In addition, we also present the weighted average results at the last row of Table 2, where the weights are determined according to the number of samples in the datasets. The larger the number of samples, the greater the weight. Due to the differences in the number of samples in the datasets involved in the simulation experiment, the results obtained by weighted average are more convincing. The proposed MeDetection model obtains the best performance with 91.8% weighted average recognition accuracy.

**Table 2:** The comparison results between MeDetection model and other state-of-the-arts for the detection tasks of MVTec (Bergmann et al., 2020b) datasets in terms of AUROC.

Tasks	GANomaly	1-NN	DOCC	PatchSVDD	U-Student	MeDetection	(20-shot)
Carpet	69.9	81.1	90.6	92.9	95.3	**99.9**	96.8
Grid	70.8	55.7	52.4	94.6	**98.7**	95.3	80.8
Leather	84.2	90.3	78.3	90.9	93.4	**100**	98.2
Tile	79.4	96.9	96.5	**97.8**	95.8	95.6	91.9
Wood	83.4	93.4	91.6	96.5	95.5	**97.3**	89.4
Bottle	89.2	98.7	**99.6**	98.6	96.7	97.1	92.1
Cable	75.7	88.5	**90.9**	90.3	82.3	82.8	77.8
Capsule	73.2	71.1	91.0	76.7	92.8	**95.1**	90.8
Hazelnut	78.5	**97.9**	95.0	92.0	91.4	95.5	92.1
Metal nut	70.0	76.7	85.2	**94.0**	**94.0**	92.3	80.3
Pill	74.3	83.7	80.4	86.1	86.7	**97.6**	86.8
Toothbrush	65.3	67.0	96.4	**100**	87.4	87.9	71.9
Screw	74.6	67.0	**86.5**	81.3	83.6	75.5	60.5
Transistor	65.3	91.9	90.8	91.5	**98.6**	94.6	86.6
Zipper	74.5	88.6	92.4	97.9	95.8	**99.9**	91.1
Avg	76.2	83.9	87.9	92.1	92.5	**94.0**	85.6
Weighted avg	74.0	82.0	85.7	89.3	90.4	**91.8**	84.3

**Note:**

Best results are in bold, and second best underlined. The last column (20-shot) denotes only 20 images are applied to train the model. An average score over all tasks is also reported at the last row (Avg). All results are presented in percentage.

In addition, challenges are attached to the MeDetection model, and only 20 images in each training task participate in model training. The simulation results are shown in the last column of Table 2. The MeDetection model achieves suboptimal AUROC on three tasks (“carpet”, “carpet” and “pill”), and the average AUROC is higher than GANomaly (Akcay, Atapour-Abarghouei & Breckon, 2018) and 1-NN (Nazare, de Mello & Ponti, 2018) methods. It is worth mentioning that, the MeDetction model exhibits relatively weak recognition performance in the task of “screw”. The MeDetection model uses differential features as input, which has high requirements for target location alignment in dual channels. However, the orientation of the screws in the “screw” dataset are placed randomly as shown in Fig. 3, making it easy to generate pseudo-features when calculating differential features. Such deficiency is one of the future research directions of the MeDetection model.

In general, MeDetection has two advantages over other models. MeDetection has the highest accuracy and good detection capability for various industrial defects. MeDetection can use a small number of samples to train the model, which reduces the training cost and has a good detection accuracy at the same time.

### Effectiveness of MAML

In order to verify the effectiveness of the MAML training training strategy in the proposed MeDetection model, we make comparison of the recognition accuracy based on random initialization of weights and MAML based weights transfer strategy. Table 3 shows the simulation results. The “Tasks” column represents the dataset used for model testing, while the remaining datasets are involved in the pre-training of MAML. The term of “random” in the column of “Weights” means that the weights used in the training of the recognition model are randomly initialized. Two absolute advantages for the MAML based training strategy for industrial defect detection can be obtained. Based on the MAML pre-trained weights, the training model has the relatively faster convergence rate. For the “fabric3” dataset, with the help of MAML pre-trained weights, the model only needs 50 epochs to converge, which is a quarter of the number of epochs of the model convergence under random weights. It is worth noting that with randomly initialized weights, the model fails to converge for the dataset of “capsule”. In addition, the MAML training strategy enables the model to improve the recognition performance in all five indicators (ACC, Recall, Precision, F1, AUROC). For the “BC defects” dataset, based on the MAML training strategy, the convergence efficiency of the model is more than doubled (450 epochs $\to$ 200 epochs). Meanwhile, the recognition performance has also been greatly improved. For the metrics of Precision, The MAML training strategy improves the recognition result by 18.8 percentage points. For the ACC, Recall, F1 and AUROC metrics, the recognition performance improved by about five percentage points. In addition, similar to Table 2, the weighted averages of AUROC scores are calculated in order to show the MeDetection model performance comprehensively. The weighted average of AUROC score of the model with randomly initialized weights is 94.7%, while the MeDetection model gets 97.6% weighted average AUROC score. We put the average results of each indicator in the last column of Table 3, which also demonstrates the performance of MeDetection model.

**Table 3:** Simulation results for the MAML based training strategy.

Tasks	Weights	Epoch	Acc (%)	Recall (%)	Precision (%)	F1 (%)	AUROC (%)	Avg (%)
Tiles	Random	260	89.1	97.5	68.1	80.2	92.1	85.4
	MAML	260	93.3	98.6	77.1	81.1	95.6	89.14
Carpet	Random	260	92.3	96.1	76.4	84.4	93.6	88.56
	MAML	200	92.4	99.9	78.1	88.2	99.9	91.7
Fabric1	Random	400	98.8	98.6	78.1	86.6	98.3	92.08
	MAML	200	99.3	98.7	96.4	97.5	99.1	98.2
Fabric2	Random	230	99.1	98.4	95.1	96.7	98.1	97.48
	MAML	150	99.8	98.6	97.8	98.1	98.2	98.5
Fabric3	Random	200	98.2	97.9	91.6	94.6	97.6	95.98
	MAML	50	99.3	99.7	97.1	98.3	99.3	98.74
Fabric4	Random	210	99.2	99.1	99.2	99.1	99.1	99.14
	MAML	180	99.8	99.3	99.3	99.3	99.2	99.38
BC defects	Random	450	83.8	89.8	77.1	82.9	86.8	84.08
	MAML	200	89.8	94.4	91.6	92.9	92.4	92.22
Sidewalk	Random	300	81.2	92.3	46.9	62.9	86.9	74.04
	MAML	300	88.3	93.4	56.4	73.2	91.2	80.5
Capsule	Random	–	86.7	76.7	80.1	78.3	84.1	81.18
	MAML	450	95.2	93.9	91.1	92.5	95.1	93.56

**Note:**

The “random” means randomly initialized weights, “Epoch” represents minimum epoch value for smooth loss variation. The symbol “–” means the model cannot converge. Avg is the average of ACC, Recall, F1 and AUROC.

**Figure 4:**
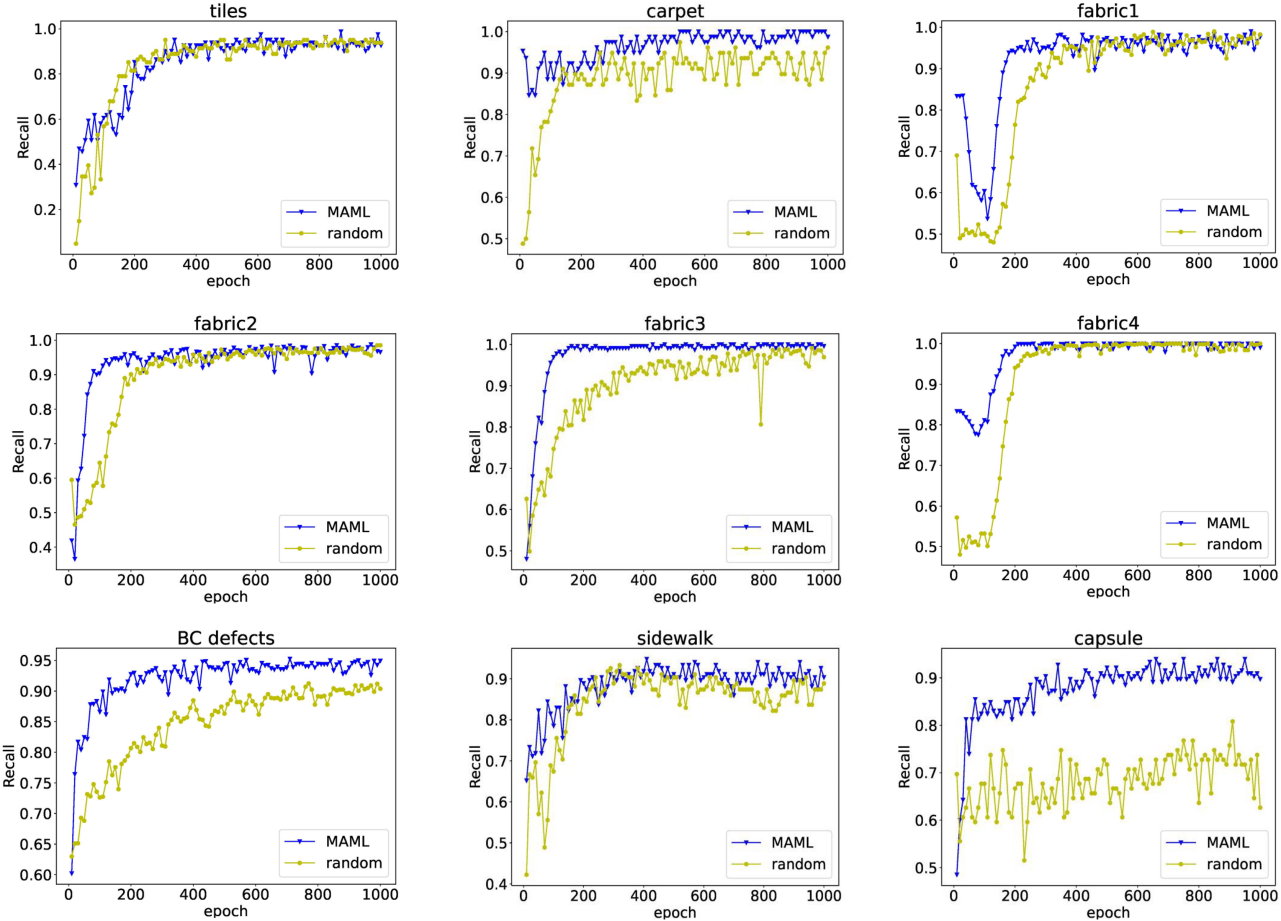
The relationship between recall rate and model training epochs. The blue line with triangle symbols shows the recall curve of each epoch when the model is fine-tuned with the initialized weights obtained from MAML training, while the yellow line with circle symbols shows the results for the model trained with random initialization weights.

For industrial defect recognition tasks, we want to reduce the missed detection rate, that is, to avoid the flow of problematic products to the market. The recall rate can clearly evaluate the missed detection of the model. Figure 4 shows the curve of recall as a function of model training epochs on nine detection tasks. Models based on MAML pretrained weights not only converge faster, but also have higher recall than those trained with random weights.

Overall, the proposed MeDetection model exhibits very advanced recognition performance for industrial defect detection. The MAML based training strategy is the corner stone of MeDetection model, which brings two advantages: MAML can accelerate the convergence speed of the model well; MAML can improve the performance of the model greatly.

### Defect location

**Figure 5:**
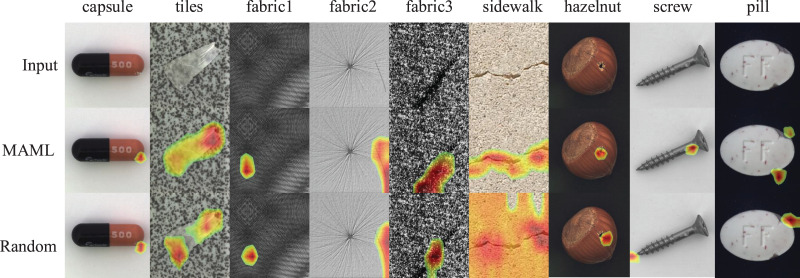
Visualization of defect location. The first row shows the original sample images with defects. The second and third rows show the localization results of the defects caused by the MAML-based training strategy and the random weight training strategy, respectively. Images taken from Bergmann et al. (2020a) (License: CC-BY-NC-SA 4.0).

The Grad-CAM algorithm (Selvaraju et al., 2017) can be used in the classification model to achieve target localization. For industrial defect detection tasks, obtaining the location information of defects is very helpful to analyze the causes of defects. Figure 5 shows the localization results of target defects, and the results of the model trained based on randomly initialized weights partipicate the comparison. The model with randomly initialized weights shows some deviations in the location of the defects, especially in the datasets of “capsule”, “fabric1” and “screw”. Additionally, for the “sidewalk” dataset, the attention response at defect locations of the model with randomly initialized weights are divergent. In contrast, the model under the MAML training strategy has stronger focusing ability on defect features and more accurate positioning accuracy.

### CA effect

The CA module is embedded into the MeDetection model for further refinement of defect features. However, whether the location and number of CA modules have an impact on model performance is an important issue. Table 4 presents the simulation results, where three embedding ways are involved in the comparative experiments. In order to fully demonstrate the effect of CA location on model performance, the Average (Avg) and Variance (Var) statistical results are also presented in Table 4. We embed the CA module in the low- or high-level of the feature extraction channel, and also take into account the effect of both embedding. Embeddings ways show differences across all datasets. Generally, when the model is embedded with CA modules at low layers, it exhibits better performance than when embedded at higher layers. The performance of the model is not stable when CA modules are embedded in the low- or high-level of the feature extraction channel. For the datasets of “fabric2” and “fabric4”, embedding multiple CA modules improves the model performance. In addition, from the Average and Variance statistical results, we can obtain the similar conclusion, that embedding the CA mudule at low lever can help the model get the best performance with the maximum average accuracy and minimum variance.

**Table 4:** Simulation results for CA modules.

Metrics	Tiles	Carpet	Fabric1	Fabric2	Fabric3	Fabric4	Cap	Sidewalk	Avg	Var
Low level	98.6	99.9	98.7	98.9	99.7	99.7	94.9	93.4	98.0	5.2
High level	98.6	99.8	98.7	98.5	99.7	99.2	94.3	93.4	97.8	5.4
Multiple	98.1	99.8	98.6	98.6	99.5	99.4	94.4	93.2	97.7	5.4

**Note:**

“Low level” means we put CA module after the first convolutional block of the 4Conv network, while “High level” means the CA module is embedded after the forth convolutional block of the 4Conv network. “Multiple” indicates that the CA modules are embedded in both the low layer and the high layer. The value of the histogram represents the average Recall metrics.

### Ablation experiment

Two feature refinement modules help the MeDetection model improve performance. The SN module further strengthens the defect features while unifying the defect representation. The CA module enhances the feature extraction ability of the model from the attention perspective. The ablation experiment is carried out to verify the roles of the two modules, and Table 5 shows the simulation results, where four datasets are taken into consideration. Obviously, the addition of the two modules greatly improves the performance of the MeDetection model. Specifically, after removing the SN module, the performance of the MeDetection model drops the most. For recognition accuracy (ACC), the performance of model without SN module drops by 18 percentage points for the dataset of “fabric4”. Undoubtedly, the performance of the model degrades the most when both the SN and CA modules are removed.

**Table 5:** Ablation experiments

Tasks	Model	Acc (%)	Recall (%)	Precision (%)	F1 (%)	AUROC (%)
BC defects	Ours	89.8	94.4	91.6	92.9	92.4
	Ours-SN	78.7	86.5	57.3	68.9	86.2
	Ours-CA	87.8	92.8	79.5	85.6	89.9
	Ours-CA-SN	76.4	84.5	54.6	66.3	84.2
Fabric4	Ours	99.2	99.1	99.2	99.1	99.1
	Ours-SN	81.1	96.6	46.4	62.6	92.4
	Ours-CA	89.2	97.1	62.3	75.9	93.8
	Ours-CA-SN	78.3	94.8	44.1	60.1	90.5
Tiles	Ours	89.1	97.5	68.1	80.2	92.1
	Ours-SN	79.6	78.7	52.4	62.9	76.5
	Ours-CA	86.6	85.4	62.7	72.3	82.4
	Ours-CA-SN	77.6	72.5	51.2	60.1	70.2
Sidewalk	Ours	81.2	92.3	46.9	62.9	86.9
	Ours-SN	77.1	83.4	41.7	55.6	82.6
	Ours-CA	77.5	86.5	42.1	56.6	83.6
	Ours-CA-SN	72.7	82.4	37.1	55.1	79.6

## Conclusion

The MeDetection model proposed in this article focuses on the rapid and adaptive visual recognition for industrial appearance defects. The biggest advantage of the MeDetection model is that the MAML training strategy is applied to the optimization of the CNN based visual detection model, so that the model can achieve satisfactory recognition performance even under the premise of limited industrial defect data sets. Simulation results (Table 5) show that our model achieves state-of-the-art performance with only 20 training epochs. In addition, MeDetection model employs the feature difference extraction module with Siamese network to convert industrial defects into feature differences, which realizes the effective unification of different types of defect features. The embedding of the coordinate attention module can make further refinement of defect features for the improvement of MeDetection model performance. Meanwhile, a visual dataset for industrial injection molded bottle cap defects, termed BC defects, is released. BC defects dataset could help to improve the benchmarks for industrial defect vision datasets. Simulation results based on BC defects dataset have verified the performance of the proposed MeDetection model.

An important research topic to further improve the performance of MeDetection model exists. Figure 5 shows that MeDetection model demonstrates unsatisfactory performance on “screw” samples in the MVTec dataset, because the samples in “screw” dataset place randomly, and misaligned features lead to spurious features in the model input. It is the main research direction in the future.

## Supplemental Information

10.7717/peerj-cs.1264/supp-1 Supplemental Information 1 Code.

## Additional Information and Declarations

### Data Availability

The following information was supplied regarding data availability: The code is available in the Supplemental Files. The data and code is available at GitHub: https://github.com/zhg-SZPT/MeDetection. The homemade dataset BC defects is available in the dataset folder. The MAML dataset contains 30 different categories of industrial products, sourced from MVTec, DAGM and our own BC defects dataset. The remaining two datasets are available at MVTec. - AD: https://www.mvtec.com/company/research/datasets/mvtec-ad/. - DAGM: https://hci.iwr.uni-heidelberg.de/content/weakly-supervised-learning-industrial-optical-inspection.
